# Crystal structure of hexa­kis­(dmpu)-di-μ_2_-hydroxido-dialuminium tetraiodide dmpu tetra­solvate [dmpu is 1,3-di­methyl­tetra­hydro­pyrimidin-2(1*H*)-one]: a centrosymmetric dinuclear aluminium complex containing AlO_5_ polyhedra

**DOI:** 10.1107/S2056989015012785

**Published:** 2015-07-08

**Authors:** Daniel Lundberg, Krzysztof Lyczko

**Affiliations:** a Department of Chemistry and Biotechnology, PO Box 7015, Swedish University of Agricultural Sciences, S-750 07 Uppsala, Sweden; b Institute of Nuclear Chemistry and Technology, Dorodna 16, PL-03-195 Warsaw, Poland

**Keywords:** crystal structure, group 13 metals, five-coordination, dmpu, space-demanding solvent

## Abstract

The structure of the title compound, [Al_2_(OH)_2_(C_6_H_12_N_2_O)_6_]I_4_·4C_6_H_12_N_2_O (systematic name: di-μ_2_-hydroxido-bis­{tris­[1,3-di­methyl­tetra­hydro­pyrimidin-2(1*H*)-one-κ*O*]aluminium} tetra­iodide 1,3-di­methyl­tetra­hydro­pyrimidin-2(1*H*)-one tetra­solvate), is composed of two Al(C_6_H_12_N_2_O)_3_ moieties linked into a centrosymmetric dinuclear unit by a pair of bridging hydroxide ions. The aluminium cations show a distorted trigonal bipyramidal AlO_5_ coordination environment formed only by monodentate ligands. The Al—O bond lengths are in the range 1.789 (2)–1.859 (2) Å (mean bond length = 1.818 Å). The non-coordinating iodide anions compensate the charge of the complex cation. The remaining solvent mol­ecules and the iodide counter-anions inter­act with the complex cation by weak non-classical C—H⋯I and C—H⋯O hydrogen bonds.

## Abstract 2

Compared to the corresponding hydrate, the space-demanding solvent ligand *N*,*N*′-di­methyl­propyl­eneurea [dmpu; systematic name: 1,3-di­methyl­tetra­hydro­pyrimidin-2(1*H*)-one] often lowers the coordination number of metal ions. For dmpu-solvated aluminium iodide, the resulting complex is a di-μ_2_-hydroxide dimer with AlO_5_ centres.

## Chemical context   

The solvent ligand *N*,*N*′-di­methyl­propyl­eneurea (dmpu; IUPAC name: 1,3-di­methyl­tetra­hydro­pyrimidin-2(1*H*)-one, C_6_H_12_N_2_O) is known to be space-demanding upon coordination. This has been shown for several different metal ions which have a lower coordination number than the corresponding hydrates (Lundberg, 2006 ▸; Lundberg *et al.*, 2010 ▸). In the boron group (group 13), the trivalent metal ions have previously been studied in dmpu solution and the solid state, 
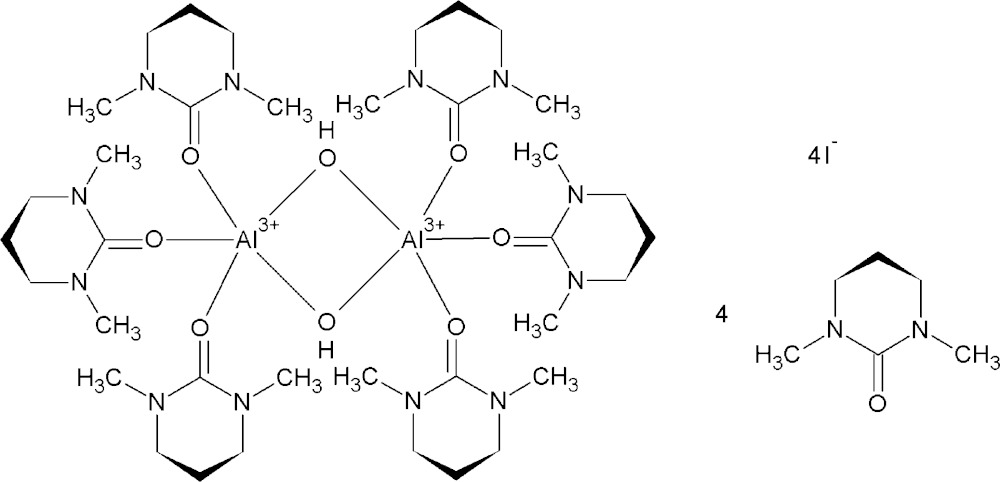
with reported crystal structures for tri­chlorido­bis­(dmpu)thallium(III) (Carmalt *et al.*, 1996 ▸) and tri­bromido­bis­(dmpu)indium(III) (Topel *et al.*, 2010 ▸). In the case of dmpu-solvated gallium(III) bromide, the gallium cation was determined to be five-coordinate in solution but crystallization was not successful despite of repeated attempts (Topel *et al.*, 2010 ▸). The title compound was prepared in an attempt to reveal the dmpu coordination for the last remaining naturally occurring trivalent group 13 metal ion, aluminium(III). Since both chloride and bromide ions are more prone to form aluminium complexes, the iodide salt was chosen as a starting material.

## Structural commentary   

**Figure 1:**
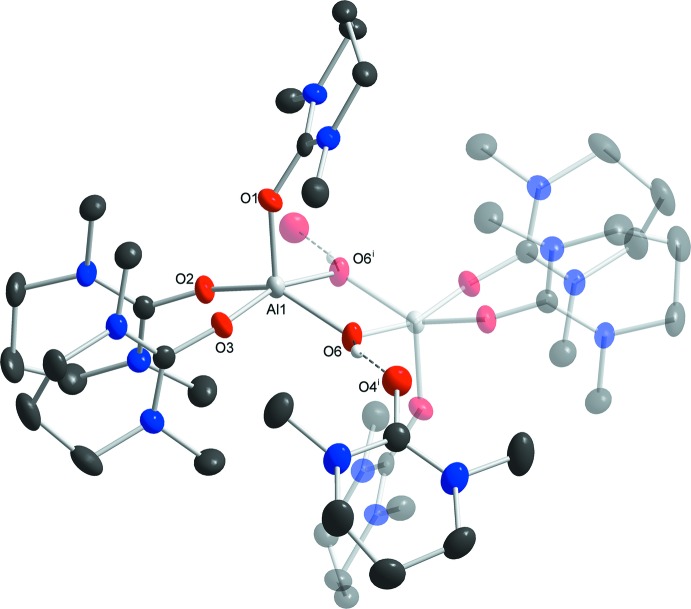
The dinuclear complex cation in the title compound, with displacement ellipsoids drawn at the 50% probability level. The hydrogen bonding from the bridging hydroxide group to the O atom (O4^i^) of one non-coordinating dmpu mol­ecule is indicated with a dashed line. Non-hydroxide H atoms have been omitted and the symmetry-related half of the complex has been shaded for clarity. [Symmetry code: (i) −*x*, 1 − *y*, 1 − *z*.]

The asymmetric unit of the title structure comprises one Al(dmpu)_3_ moiety, two dmpu solvent mol­ecules and two iodide counter anions. The dinuclear cationic aluminium complex (Fig. 1 ▸) is generated by inversion symmetry and contains two five-coordinate aluminium cations, in which each cation is coordinated by the oxygen atoms of three dmpu ligand mol­ecules and two μ_2_-bridging hydroxide ions, completing an AlO_5_ coordination sphere. The Al—O bond lengths in the Al_2_(μ_2_-OH)_2_ bridge are 1.804 (2) and 1.859 (2) Å, while the Al—O bonds to the dmpu ligand mol­ecules are 1.789 (2), 1.792 (2), and 1.846 (2) Å, respectively. The two aluminium cations are separated by 2.883 (1) Å from each other. The Al—O—C angles for the coordinating dmpu ligand mol­ecules lie in the range of 144.0 (2) to 154.7 (2)°. The dmpu ligand mol­ecules are all essentially flat with the exception of the middle propyl­ene carbon atom which is bent out of the plane with a dihedral angle of *ca* 50°.

## Supra­molecular features   

**Figure 2:**
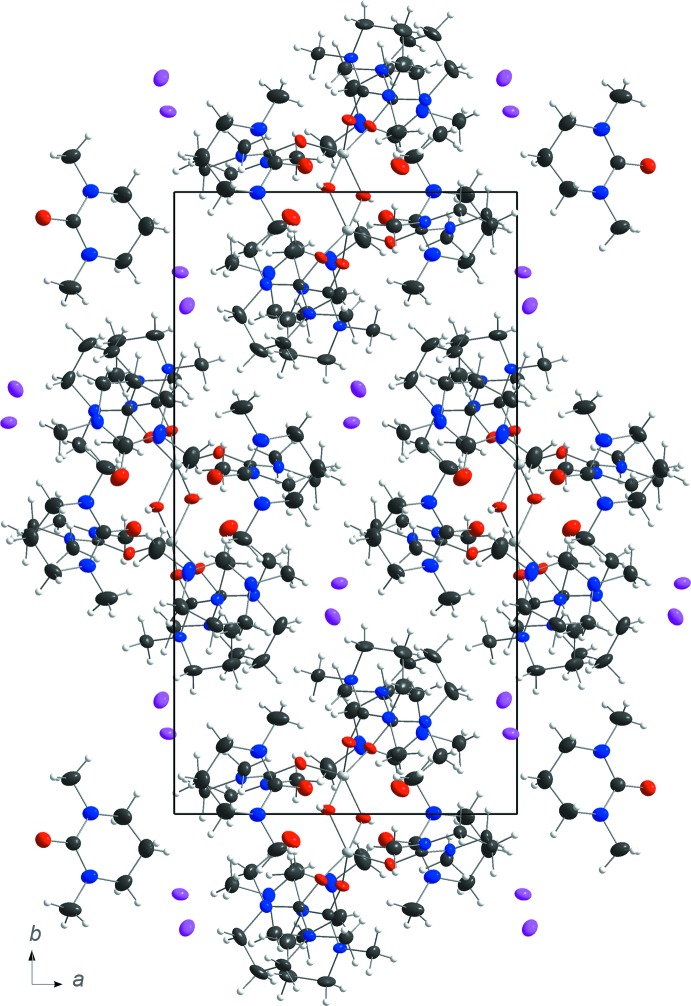
The crystal packing of the title structure in a view along [001].

In the crystal packing, the complex cations are arranged in rods parallel to [001] with the counter-anions situated between the rods (Fig. 2 ▸). The hydroxide ion forms a medium-strength O—H⋯O hydrogen bond of 2.625 (3) Å to one of the non-coordinating dmpu ligand mol­ecules, with an H⋯O—C angle for this inter­action of 134.8 (17)°. The other non-coordinating dmpu mol­ecule is stabilized by a much weaker O⋯H—C inter­action of 3.190 (5) Å. Other O⋯H—C inter­action between the moieties range from 3.404 (5)–3.561 (4) Å. The remaining positive charges on the aluminium atoms in the complex are compensated by the presence of non-coordin­ating iodide anions, which inter­act with the cationic complex by weak I⋯H—C hydrogen bonds in the range 3.932 (4)–4.070 (4) Å (Table 1 ▸).

## Database survey   

The Cambridge Structural Database (Version 2015; Groom & Allen, 2014 ▸) lists 615 structures with an AlO_4_ coordination polyhedron and 387 structures with an AlO_6_ polyhedron, but only 46 with an AlO_5_ polyhedron. Of these 46, three contain μ_2_-hydroxido bridges, including two polynuclear structures (Abrahams *et al.*, 2002 ▸; Murugavel & Kuppuswamy, 2006 ▸) and a trinuclear structure with an AlO_3_N_2_–AlO_5_–AlO_3_N_2_ motif. Another trinuclear complex with an AlO_4_–AlO_5_–AlO_4_ motif, albeit without hydroxide bridges (Pauls & Neumüller, 2000 ▸), and two different mononuclear, five-coordinate tetra­hydro­furan (thf) solvates have been reported (Karsch *et al.*, 2012 ▸). More than 50 examples of dimeric complexes with hexa­coordinate aluminium ions with similar bridging between aluminium have been reported.

Urea solvated aluminium perchlorate was structurally determined by Mooy *et al.* (1974 ▸) as a hexa­coordinate, homoleptic complex. Homoleptic hexa­coordination is also found in other common, non-aqueous *O*-donor solvents, including di­methyl­sulfoxide (dmso) solvated aluminium chloride (Boström *et al.*, 2003 ▸), hexa­iso­thio­cyanato­aluminium (Gumbris *et al.*, 2012 ▸), iodide (Molla-Abbassi *et al.*, 2003 ▸), and perchlorate (Chan *et al.*, 2004 ▸), as well as *N*,*N*-di­methyl­formamide (dmf) solvated aluminium hexa­chlorido­technate chloride (Benz *et al.*, 2015 ▸), perchlorate (Suzuki & Ishiguro, 1998 ▸), and tribromide (Bekaert *et al.*, 2002 ▸), and the *N*,*N*-di­methyl­acetamide (dma) solvated aluminium perchlorate (Suzuki & Ishiguro, 2006 ▸). One homoleptic, tetra­coordinate aluminium ion has been reported by Engesser *et al.* (2012 ▸) with an anionic *O*-donor ligand.

## Synthesis and crystallization   

**Figure 3:**
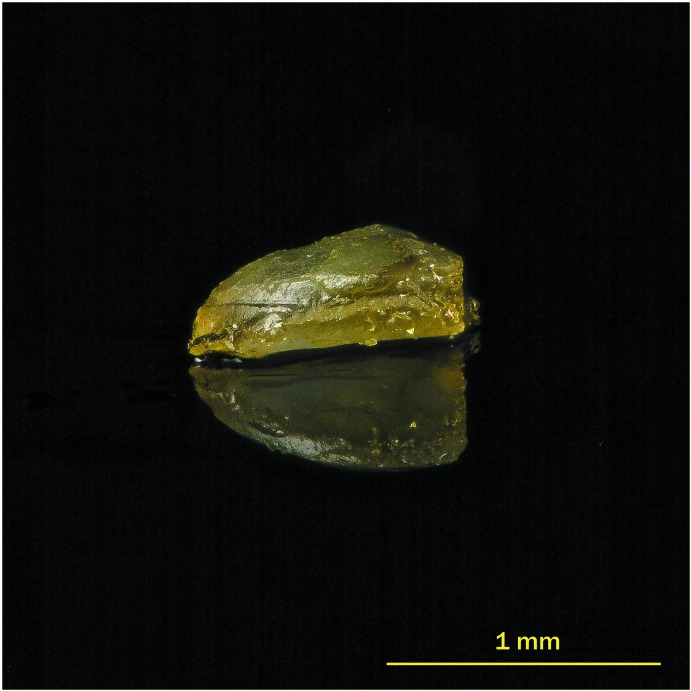
High-resolution photograph of another, partially crystalline sample of the title compound. Multiple exposures were stacked for an increased depth of field.

The title compound was prepared by dissolving anhydrous aluminium(III) iodide (Sigma–Aldrich) in distilled dmpu in a glass vial, and subsequently heated in an oil bath to approximately 323 K, and then allowed to cool while still in the oil bath. After cooling to room temperature, the sample was refrigerated (277 K) for several weeks to allow for crystal growth. The presence of hy­droxide ions in the title compound was most likely caused during preparation of the mother liquor. It appears possible that with additional precautions, a hydroxide-free compound might be obtained. A part of the solid was photographed in detail at ambient room temperature (Fig. 3 ▸), whereas attempts to study smaller crystals failed, presumably due to the hygroscopicity of the material.

## Refinement   

Hydrogen atoms bonded to carbon atoms were placed in calculated positions with C—H = 0.98 (meth­yl) or 0.99 Å (methyl­ene) and refined isotropically using a riding model with *U*_iso_(H) equal to 1.5*U*_eq_(C) or 1.2*U*_eq_(C) for methyl and methyl­ene hydrogen atoms, respectively. The hydrogen atom of the hydroxide group was located in a difference map and its position and *U*_iso_ value were freely refined. Crystal data, data collection and structure refinement details are summarized in Table 2 ▸.

## Supplementary Material

Crystal structure: contains datablock(s) I. DOI: 10.1107/S2056989015012785/wm5176sup1.cif

Structure factors: contains datablock(s) I. DOI: 10.1107/S2056989015012785/wm5176Isup2.hkl

CCDC reference: 1410078

Additional supporting information:  crystallographic information; 3D view; checkCIF report

## Figures and Tables

**Table 1:** Hydrogen-bond geometry (, )

*D*H*A*	*D*H	H*A*	*D* *A*	*D*H*A*
O6H6O4^i^	0.73(5)	1.91(5)	2.625(3)	167(5)
C5H5*B*I2	0.98	3.01	3.987(3)	172
C6H6*B*O5^ii^	0.98	2.21	3.190(4)	174
C12H12*A*O1	0.98	2.59	3.561(4)	173
C12H12*B*I1^iii^	0.98	3.09	4.051(3)	167
C14H14*A*I2^iv^	0.99	3.15	4.070(4)	156
C17H17*B*I1^iv^	0.98	3.05	4.015(4)	169
C16H16*A*I1^iii^	0.99	3.11	3.932(4)	141
C24H24*A*O3^i^	0.98	2.57	3.482(5)	154
C28H28*B*I2^v^	0.99	3.09	3.981(4)	150
C30H30*A*O5^vi^	0.98	2.57	3.404(5)	143

Symmetry codes: (i) 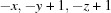; (ii) 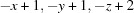; (iii) 
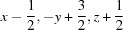
; (iv) 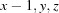; (v) 
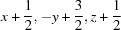
; (vi) 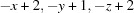.

**Table 2:** Experimental details

Crystal data	
Chemical formula	[Al_2_(OH)_2_(C_6_H_12_N_2_O)_6_]I_4_4C_6_H_12_N_2_O
*M*_r_	1877.33
Crystal system, space group	Monoclinic, *P*2_1_/*n*
Temperature (K)	100
*a*, *b*, *c* ()	13.9120(2), 22.6152(2), 14.4875(3)
()	116.331(2)
*V* (^3^)	4085.16(12)
*Z*	2
Radiation type	Cu *K*
(mm^1^)	12.72
Crystal size (mm)	0.20 0.16 0.14

Data collection	
Diffractometer	Agilent SuperNova Dual Source diffractometer with an Eos detector
Absorption correction	Multi-scan (*CrysAlis PRO*; Agilent, 2014 ▸)
*T*_min_, *T*_max_	0.411, 1.000
No. of measured, independent and observed [*I* > 2(*I*)] reflections	75993, 7114, 6779
*R*_int_	0.040
(sin /)_max_ (^1^)	0.593

Refinement	
*R*[*F* ^2^ > 2(*F* ^2^)], *wR*(*F* ^2^), *S*	0.030, 0.078, 1.10
No. of reflections	7114
No. of parameters	456
H-atom treatment	H atoms treated by a mixture of independent and constrained refinement
_max_, _min_ (e ^3^)	1.20, 1.13

Computer programs: *CrysAlis PRO* (Agilent, 2014 ▸), *SHELXS97* (Sheldrick, 2008 ▸), *SHELXL2014* (Sheldrick, 2015 ▸) and *DIAMOND* (Crystal Impact, 2001 ▸).

## supplementary crystallographic information

### Crystal data

**Table:**

[Al_2_(OH)_2_(C_6_H_12_N_2_O)_6_]I_4_·4C_6_H_12_N2O	*F*(000) = 1912
*M**_r_* = 1877.33	*D*_x_ = 1.526 Mg m^−^^3^
Monoclinic, *P*2_1_/*n*	Cu *K*α radiation, λ = 1.54184 Å
*a* = 13.9120 (2) Å	Cell parameters from 30242 reflections
*b* = 22.6152 (2) Å	θ = 3.9–69.2°
*c* = 14.4875 (3) Å	µ = 12.72 mm^−^^1^
β = 116.331 (2)°	*T* = 100 K
*V* = 4085.16 (12) Å^3^	Block, yellow
*Z* = 2	0.20 × 0.16 × 0.14 mm

### Data collection

**Table:**

Agilent SuperNova Dual Source diffractometer with an Eos detector	7114 independent reflections
Radiation source: SuperNova (Cu) X-ray Source	6779 reflections with *I* > 2σ(*I*)
Detector resolution: 16.0131 pixels mm^-1^	*R*_int_ = 0.040
ω scans	θ_max_ = 66.0°, θ_min_ = 3.7°
Absorption correction: multi-scan (*CrysAlis PRO*; Agilent, 2014)	*h* = −16→16
*T*_min_ = 0.411, *T*_max_ = 1.000	*k* = −26→26
75993 measured reflections	*l* = −17→17

### Refinement

**Table:**

Refinement on *F*^2^	0 restraints
Least-squares matrix: full	Hydrogen site location: mixed
*R*[*F*^2^ > 2σ(*F*^2^)] = 0.030	H atoms treated by a mixture of independent and constrained refinement
*wR*(*F*^2^) = 0.078	*w* = 1/[σ^2^(*F*_o_^2^) + (0.0364*P*)^2^ + 6.5832*P*] where *P* = (*F*_o_^2^ + 2*F*_c_^2^)/3
*S* = 1.10	(Δ/σ)_max_ = 0.002
7114 reflections	Δρ_max_ = 1.20 e Å^−^^3^
456 parameters	Δρ_min_ = −1.13 e Å^−^^3^

### Special details

**Table:**

Geometry. All e.s.d.'s (except the e.s.d. in the dihedral angle between two l.s. planes) are estimated using the full covariance matrix. The cell e.s.d.'s are taken into account individually in the estimation of e.s.d.'s in distances, angles and torsion angles; correlations between e.s.d.'s in cell parameters are only used when they are defined by crystal symmetry. An approximate (isotropic) treatment of cell e.s.d.'s is used for estimating e.s.d.'s involving l.s. planes.

### Fractional atomic coordinates and isotropic or equivalent isotropic displacement parameters (Å^2^)

**Table:**

	*x*	*y*	*z*	*U*_iso_*/*U*_eq_	
I1	0.51772 (2)	0.62910 (2)	0.13190 (2)	0.03025 (7)	
I2	0.53752 (2)	0.68312 (2)	0.63215 (2)	0.03565 (8)	
Al1	0.00668 (7)	0.56180 (4)	0.52651 (7)	0.01970 (18)	
O1	0.13114 (16)	0.58133 (9)	0.63291 (16)	0.0250 (5)	
O3	−0.07158 (16)	0.60555 (9)	0.57705 (16)	0.0240 (4)	
N9	0.7761 (2)	0.63074 (12)	0.9445 (2)	0.0298 (6)	
N2	0.2309 (2)	0.54816 (11)	0.7944 (2)	0.0251 (5)	
N1	0.3048 (2)	0.55782 (11)	0.6791 (2)	0.0252 (5)	
C1	0.2217 (2)	0.56185 (12)	0.7019 (2)	0.0218 (6)	
O2	−0.00991 (17)	0.61562 (9)	0.42976 (17)	0.0261 (5)	
N4	−0.0161 (2)	0.71546 (10)	0.41492 (19)	0.0230 (5)	
N8	0.2568 (2)	0.49894 (13)	0.3074 (2)	0.0338 (7)	
C7	−0.0504 (2)	0.66163 (13)	0.3756 (2)	0.0194 (6)	
O4	0.11822 (19)	0.54165 (10)	0.32421 (19)	0.0339 (5)	
C12	0.0803 (3)	0.72256 (14)	0.5114 (2)	0.0277 (7)	
H12A	0.0955	0.6856	0.5506	0.042*	
H12B	0.0694	0.7544	0.5516	0.042*	
H12C	0.1408	0.7324	0.4969	0.042*	
N5	−0.2321 (2)	0.64829 (11)	0.4770 (2)	0.0259 (6)	
N6	−0.1056 (2)	0.69603 (11)	0.6212 (2)	0.0241 (5)	
N3	−0.1258 (2)	0.65501 (11)	0.2794 (2)	0.0245 (5)	
C17	−0.2762 (3)	0.59303 (17)	0.4251 (3)	0.0413 (9)	
H17A	−0.2177	0.5654	0.4364	0.062*	
H17B	−0.3179	0.6002	0.3511	0.062*	
H17C	−0.3230	0.5760	0.4526	0.062*	
O5	0.8410 (2)	0.54111 (12)	1.0114 (2)	0.0441 (6)	
C13	−0.1357 (2)	0.64952 (13)	0.5582 (2)	0.0201 (6)	
C5	0.2954 (3)	0.57220 (15)	0.5773 (3)	0.0313 (7)	
H5A	0.3051	0.5362	0.5447	0.047*	
H5B	0.3505	0.6012	0.5840	0.047*	
H5C	0.2242	0.5889	0.5349	0.047*	
C6	0.1414 (3)	0.55467 (15)	0.8202 (3)	0.0311 (7)	
H6A	0.1458	0.5933	0.8525	0.047*	
H6B	0.1446	0.5233	0.8681	0.047*	
H6C	0.0737	0.5518	0.7573	0.047*	
N7	0.2533 (2)	0.60067 (13)	0.3315 (2)	0.0342 (7)	
N10	0.9575 (2)	0.61058 (15)	1.0066 (2)	0.0411 (7)	
C20	0.3530 (3)	0.61090 (17)	0.3229 (3)	0.0410 (9)	
H20A	0.3949	0.6425	0.3715	0.049*	
H20B	0.3360	0.6242	0.2523	0.049*	
C18	0.0012 (3)	0.69860 (14)	0.7087 (3)	0.0293 (7)	
H18A	−0.0035	0.6866	0.7715	0.044*	
H18B	0.0288	0.7391	0.7167	0.044*	
H18C	0.0497	0.6719	0.6963	0.044*	
C14	−0.3091 (3)	0.69760 (17)	0.4505 (3)	0.0367 (8)	
H14A	−0.3642	0.6882	0.4741	0.044*	
H14B	−0.3457	0.7027	0.3748	0.044*	
C8	−0.1683 (3)	0.70498 (16)	0.2085 (3)	0.0360 (8)	
H8A	−0.2426	0.6963	0.1569	0.043*	
H8B	−0.1241	0.7113	0.1714	0.043*	
C15	−0.2530 (3)	0.75435 (16)	0.5002 (3)	0.0403 (9)	
H15A	−0.2094	0.7686	0.4661	0.048*	
H15B	−0.3066	0.7851	0.4927	0.048*	
C27	0.8817 (3)	0.69576 (16)	0.8932 (3)	0.0377 (8)	
H27A	0.8970	0.7377	0.8856	0.045*	
H27B	0.8598	0.6759	0.8260	0.045*	
C26	0.7926 (3)	0.69190 (15)	0.9246 (3)	0.0328 (7)	
H26A	0.8109	0.7158	0.9874	0.039*	
H26B	0.7257	0.7080	0.8691	0.039*	
C3	0.4264 (3)	0.55167 (14)	0.8624 (3)	0.0293 (7)	
H3A	0.4940	0.5336	0.9132	0.035*	
H3B	0.4324	0.5951	0.8725	0.035*	
C19	0.2059 (3)	0.54700 (14)	0.3204 (3)	0.0275 (7)	
C16	−0.1821 (3)	0.74310 (14)	0.6123 (3)	0.0327 (7)	
H16A	−0.1429	0.7797	0.6455	0.039*	
H16B	−0.2260	0.7309	0.6472	0.039*	
C28	0.9807 (3)	0.66668 (17)	0.9733 (3)	0.0413 (9)	
H28A	1.0326	0.6605	0.9446	0.050*	
H28B	1.0144	0.6933	1.0335	0.050*	
C25	0.8574 (3)	0.59161 (16)	0.9887 (3)	0.0329 (7)	
C29	0.6688 (3)	0.61365 (16)	0.9271 (3)	0.0324 (7)	
H29A	0.6521	0.6318	0.9797	0.049*	
H29B	0.6649	0.5705	0.9310	0.049*	
H29C	0.6168	0.6271	0.8587	0.049*	
C4	0.3343 (2)	0.52907 (14)	0.8798 (3)	0.0284 (7)	
H4A	0.3370	0.4854	0.8837	0.034*	
H4B	0.3400	0.5445	0.9460	0.034*	
C2	0.4107 (2)	0.53720 (15)	0.7553 (3)	0.0305 (7)	
H2A	0.4675	0.5565	0.7424	0.037*	
H2B	0.4163	0.4939	0.7485	0.037*	
C21	0.4188 (3)	0.55523 (19)	0.3467 (4)	0.0479 (10)	
H21A	0.4791	0.5607	0.3289	0.057*	
H21B	0.4489	0.5465	0.4213	0.057*	
C24	0.1992 (3)	0.44301 (16)	0.2775 (3)	0.0406 (9)	
H24A	0.1483	0.4403	0.3072	0.061*	
H24B	0.2504	0.4102	0.3028	0.061*	
H24C	0.1601	0.4409	0.2022	0.061*	
C11	−0.1538 (3)	0.59608 (16)	0.2335 (3)	0.0401 (9)	
H11A	−0.0989	0.5826	0.2134	0.060*	
H11B	−0.2235	0.5977	0.1726	0.060*	
H11C	−0.1579	0.5685	0.2839	0.060*	
C30	1.0498 (3)	0.5726 (2)	1.0638 (4)	0.0552 (11)	
H30A	1.0688	0.5512	1.0154	0.083*	
H30B	1.0319	0.5443	1.1049	0.083*	
H30C	1.1107	0.5969	1.1095	0.083*	
C9	−0.1671 (3)	0.75993 (15)	0.2679 (3)	0.0423 (9)	
H9A	−0.1890	0.7945	0.2211	0.051*	
H9B	−0.2187	0.7556	0.2975	0.051*	
C10	−0.0559 (3)	0.76947 (14)	0.3529 (3)	0.0365 (8)	
H10A	−0.0069	0.7806	0.3228	0.044*	
H10B	−0.0570	0.8023	0.3976	0.044*	
C22	0.3500 (3)	0.50443 (17)	0.2862 (4)	0.0467 (10)	
H22A	0.3258	0.5112	0.2117	0.056*	
H22B	0.3922	0.4674	0.3055	0.056*	
C23	0.1972 (4)	0.65363 (17)	0.3377 (4)	0.0493 (11)	
H23A	0.2429	0.6759	0.3998	0.074*	
H23B	0.1306	0.6424	0.3407	0.074*	
H23C	0.1804	0.6783	0.2768	0.074*	
O6	−0.05183 (18)	0.49339 (9)	0.54105 (19)	0.0250 (5)	
H6	−0.077 (4)	0.487 (2)	0.575 (4)	0.051 (14)*	

### Atomic displacement parameters (Å^2^)

**Table:**

	*U*^11^	*U*^22^	*U*^33^	*U*^12^	*U*^13^	*U*^23^
I1	0.04045 (13)	0.02150 (11)	0.02735 (12)	0.00304 (8)	0.01372 (10)	0.00282 (7)
I2	0.03502 (13)	0.04120 (13)	0.03083 (13)	−0.00851 (9)	0.01468 (10)	0.00502 (9)
Al1	0.0201 (4)	0.0147 (4)	0.0245 (5)	0.0025 (3)	0.0100 (4)	0.0015 (3)
O1	0.0197 (10)	0.0256 (11)	0.0251 (11)	0.0052 (8)	0.0056 (9)	0.0025 (9)
O3	0.0236 (10)	0.0202 (10)	0.0275 (12)	0.0074 (8)	0.0108 (9)	0.0007 (9)
N9	0.0238 (14)	0.0334 (15)	0.0315 (16)	0.0003 (11)	0.0117 (12)	0.0067 (12)
N2	0.0236 (13)	0.0222 (13)	0.0254 (14)	−0.0010 (10)	0.0072 (11)	0.0015 (11)
N1	0.0178 (12)	0.0244 (13)	0.0290 (14)	0.0001 (10)	0.0064 (11)	0.0013 (11)
C1	0.0244 (15)	0.0104 (13)	0.0232 (16)	−0.0008 (11)	0.0037 (13)	−0.0004 (11)
O2	0.0300 (11)	0.0178 (10)	0.0282 (12)	0.0045 (9)	0.0109 (10)	0.0059 (9)
N4	0.0282 (13)	0.0160 (12)	0.0200 (13)	−0.0007 (10)	0.0064 (11)	0.0000 (10)
N8	0.0378 (16)	0.0288 (15)	0.0464 (18)	−0.0013 (12)	0.0292 (15)	−0.0037 (13)
C7	0.0212 (14)	0.0189 (14)	0.0223 (15)	0.0017 (11)	0.0135 (13)	0.0023 (12)
O4	0.0349 (13)	0.0353 (13)	0.0415 (14)	0.0014 (10)	0.0261 (11)	0.0045 (11)
C12	0.0287 (16)	0.0257 (16)	0.0249 (17)	−0.0043 (13)	0.0085 (14)	−0.0061 (13)
N5	0.0233 (13)	0.0242 (13)	0.0285 (14)	0.0029 (11)	0.0099 (11)	0.0028 (11)
N6	0.0260 (13)	0.0184 (12)	0.0303 (14)	0.0018 (10)	0.0145 (12)	−0.0012 (11)
N3	0.0251 (13)	0.0220 (13)	0.0209 (13)	−0.0004 (10)	0.0053 (11)	−0.0014 (10)
C17	0.0308 (18)	0.037 (2)	0.041 (2)	−0.0051 (15)	0.0025 (16)	−0.0028 (17)
O5	0.0497 (16)	0.0426 (15)	0.0432 (16)	0.0126 (12)	0.0236 (13)	0.0164 (12)
C13	0.0209 (14)	0.0201 (14)	0.0239 (15)	−0.0001 (11)	0.0142 (13)	0.0033 (12)
C5	0.0252 (16)	0.0335 (18)	0.0347 (19)	−0.0034 (13)	0.0128 (14)	0.0026 (14)
C6	0.0355 (18)	0.0298 (17)	0.0285 (18)	0.0015 (14)	0.0146 (15)	0.0014 (14)
N7	0.0409 (16)	0.0289 (15)	0.0452 (18)	−0.0031 (12)	0.0304 (15)	−0.0029 (13)
N10	0.0280 (15)	0.055 (2)	0.0359 (17)	0.0078 (14)	0.0106 (13)	0.0061 (15)
C20	0.044 (2)	0.0361 (19)	0.054 (2)	−0.0123 (16)	0.0319 (19)	−0.0053 (17)
C18	0.0308 (17)	0.0279 (16)	0.0266 (17)	−0.0025 (13)	0.0105 (14)	−0.0029 (13)
C14	0.0291 (17)	0.048 (2)	0.0304 (19)	0.0161 (16)	0.0104 (15)	0.0080 (16)
C8	0.0393 (19)	0.0365 (19)	0.0217 (17)	−0.0014 (15)	0.0041 (15)	0.0080 (14)
C15	0.048 (2)	0.0300 (18)	0.047 (2)	0.0204 (16)	0.0247 (19)	0.0112 (16)
C27	0.044 (2)	0.0333 (19)	0.039 (2)	−0.0115 (16)	0.0213 (17)	−0.0041 (16)
C26	0.0345 (18)	0.0314 (17)	0.0288 (18)	−0.0045 (14)	0.0106 (15)	0.0019 (14)
C3	0.0224 (15)	0.0243 (16)	0.0317 (18)	−0.0015 (12)	0.0032 (14)	0.0001 (13)
C19	0.0329 (17)	0.0280 (16)	0.0267 (17)	0.0003 (13)	0.0179 (14)	0.0006 (13)
C16	0.0379 (18)	0.0230 (16)	0.045 (2)	0.0067 (14)	0.0250 (17)	−0.0010 (14)
C28	0.0352 (19)	0.042 (2)	0.050 (2)	−0.0098 (16)	0.0223 (18)	−0.0104 (18)
C25	0.0358 (18)	0.039 (2)	0.0252 (17)	0.0030 (15)	0.0150 (15)	0.0072 (15)
C29	0.0289 (17)	0.0383 (18)	0.0306 (18)	−0.0023 (14)	0.0137 (15)	0.0035 (15)
C4	0.0254 (16)	0.0258 (16)	0.0260 (17)	0.0012 (13)	0.0043 (13)	0.0041 (13)
C2	0.0196 (15)	0.0310 (17)	0.0352 (19)	0.0003 (13)	0.0070 (14)	0.0000 (14)
C21	0.035 (2)	0.052 (2)	0.066 (3)	−0.0025 (17)	0.031 (2)	0.002 (2)
C24	0.053 (2)	0.0293 (18)	0.049 (2)	−0.0092 (16)	0.031 (2)	−0.0088 (16)
C11	0.042 (2)	0.0320 (19)	0.034 (2)	−0.0023 (15)	0.0050 (17)	−0.0084 (15)
C30	0.040 (2)	0.069 (3)	0.052 (3)	0.013 (2)	0.016 (2)	0.013 (2)
C9	0.049 (2)	0.0262 (18)	0.038 (2)	0.0098 (16)	0.0061 (18)	0.0123 (15)
C10	0.050 (2)	0.0159 (15)	0.035 (2)	0.0000 (14)	0.0113 (17)	0.0055 (14)
C22	0.050 (2)	0.038 (2)	0.073 (3)	0.0029 (17)	0.047 (2)	−0.001 (2)
C23	0.072 (3)	0.0280 (19)	0.070 (3)	0.0047 (18)	0.052 (3)	0.0048 (18)
O6	0.0320 (12)	0.0162 (10)	0.0356 (13)	0.0016 (8)	0.0230 (11)	0.0015 (9)

### Geometric parameters (Å, º)

**Table:**

Al1—O1	1.789 (2)	C18—H18B	0.9800
Al1—O2	1.792 (2)	C18—H18C	0.9800
Al1—O6	1.804 (2)	C14—C15	1.509 (5)
Al1—O3	1.846 (2)	C14—H14A	0.9900
Al1—O6^i^	1.859 (2)	C14—H14B	0.9900
Al1—Al1^i^	2.8831 (16)	C8—C9	1.507 (5)
O1—C1	1.290 (4)	C8—H8A	0.9900
O3—C13	1.282 (4)	C8—H8B	0.9900
N9—C25	1.353 (4)	C15—C16	1.501 (5)
N9—C26	1.452 (4)	C15—H15A	0.9900
N9—C29	1.452 (4)	C15—H15B	0.9900
N2—C1	1.324 (4)	C27—C26	1.500 (5)
N2—C6	1.457 (4)	C27—C28	1.502 (6)
N2—C4	1.485 (4)	C27—H27A	0.9900
N1—C1	1.340 (4)	C27—H27B	0.9900
N1—C5	1.458 (4)	C26—H26A	0.9900
N1—C2	1.471 (4)	C26—H26B	0.9900
O2—C7	1.274 (4)	C3—C4	1.502 (5)
N4—C7	1.339 (4)	C3—C2	1.503 (5)
N4—C12	1.454 (4)	C3—H3A	0.9900
N4—C10	1.471 (4)	C3—H3B	0.9900
N8—C19	1.356 (4)	C16—H16A	0.9900
N8—C24	1.457 (4)	C16—H16B	0.9900
N8—C22	1.464 (4)	C28—H28A	0.9900
C7—N3	1.330 (4)	C28—H28B	0.9900
O4—C19	1.251 (4)	C29—H29A	0.9800
C12—H12A	0.9800	C29—H29B	0.9800
C12—H12B	0.9800	C29—H29C	0.9800
C12—H12C	0.9800	C4—H4A	0.9900
N5—C13	1.336 (4)	C4—H4B	0.9900
N5—C17	1.447 (4)	C2—H2A	0.9900
N5—C14	1.475 (4)	C2—H2B	0.9900
N6—C13	1.332 (4)	C21—C22	1.503 (6)
N6—C18	1.465 (4)	C21—H21A	0.9900
N6—C16	1.470 (4)	C21—H21B	0.9900
N3—C11	1.463 (4)	C24—H24A	0.9800
N3—C8	1.464 (4)	C24—H24B	0.9800
C17—H17A	0.9800	C24—H24C	0.9800
C17—H17B	0.9800	C11—H11A	0.9800
C17—H17C	0.9800	C11—H11B	0.9800
O5—C25	1.237 (4)	C11—H11C	0.9800
C5—H5A	0.9800	C30—H30A	0.9800
C5—H5B	0.9800	C30—H30B	0.9800
C5—H5C	0.9800	C30—H30C	0.9800
C6—H6A	0.9800	C9—C10	1.506 (5)
C6—H6B	0.9800	C9—H9A	0.9900
C6—H6C	0.9800	C9—H9B	0.9900
N7—C19	1.356 (4)	C10—H10A	0.9900
N7—C23	1.454 (5)	C10—H10B	0.9900
N7—C20	1.465 (4)	C22—H22A	0.9900
N10—C25	1.368 (5)	C22—H22B	0.9900
N10—C28	1.444 (5)	C23—H23A	0.9800
N10—C30	1.459 (5)	C23—H23B	0.9800
C20—C21	1.504 (6)	C23—H23C	0.9800
C20—H20A	0.9900	O6—Al1^i^	1.859 (2)
C20—H20B	0.9900	O6—H6	0.73 (5)
C18—H18A	0.9800		
			
O1—Al1—O2	104.24 (11)	C16—C15—H15B	109.9
O1—Al1—O6	115.18 (11)	C14—C15—H15B	109.9
O2—Al1—O6	139.96 (12)	H15A—C15—H15B	108.3
O1—Al1—O3	92.39 (10)	C26—C27—C28	109.8 (3)
O2—Al1—O3	92.99 (10)	C26—C27—H27A	109.7
O6—Al1—O3	92.13 (10)	C28—C27—H27A	109.7
O1—Al1—O6^i^	101.27 (11)	C26—C27—H27B	109.7
O2—Al1—O6^i^	90.04 (11)	C28—C27—H27B	109.7
O6—Al1—O6^i^	76.16 (12)	H27A—C27—H27B	108.2
O3—Al1—O6^i^	164.83 (11)	N9—C26—C27	109.9 (3)
O1—Al1—Al1^i^	113.09 (8)	N9—C26—H26A	109.7
O2—Al1—Al1^i^	118.65 (9)	C27—C26—H26A	109.7
O6—Al1—Al1^i^	38.76 (7)	N9—C26—H26B	109.7
O3—Al1—Al1^i^	130.21 (8)	C27—C26—H26B	109.7
O6^i^—Al1—Al1^i^	37.40 (7)	H26A—C26—H26B	108.2
C1—O1—Al1	145.4 (2)	C4—C3—C2	110.8 (3)
C13—O3—Al1	144.0 (2)	C4—C3—H3A	109.5
C25—N9—C26	123.0 (3)	C2—C3—H3A	109.5
C25—N9—C29	119.2 (3)	C4—C3—H3B	109.5
C26—N9—C29	117.4 (3)	C2—C3—H3B	109.5
C1—N2—C6	121.6 (3)	H3A—C3—H3B	108.1
C1—N2—C4	122.3 (3)	O4—C19—N8	120.6 (3)
C6—N2—C4	116.0 (3)	O4—C19—N7	120.9 (3)
C1—N1—C5	122.3 (3)	N8—C19—N7	118.5 (3)
C1—N1—C2	121.6 (3)	N6—C16—C15	108.7 (3)
C5—N1—C2	116.1 (3)	N6—C16—H16A	109.9
O1—C1—N2	119.2 (3)	C15—C16—H16A	109.9
O1—C1—N1	119.0 (3)	N6—C16—H16B	109.9
N2—C1—N1	121.7 (3)	C15—C16—H16B	109.9
C7—O2—Al1	154.7 (2)	H16A—C16—H16B	108.3
C7—N4—C12	120.8 (2)	N10—C28—C27	112.2 (3)
C7—N4—C10	121.9 (3)	N10—C28—H28A	109.2
C12—N4—C10	115.6 (2)	C27—C28—H28A	109.2
C19—N8—C24	119.0 (3)	N10—C28—H28B	109.2
C19—N8—C22	121.8 (3)	C27—C28—H28B	109.2
C24—N8—C22	115.7 (3)	H28A—C28—H28B	107.9
O2—C7—N3	118.8 (3)	O5—C25—N9	121.0 (3)
O2—C7—N4	120.3 (3)	O5—C25—N10	122.1 (3)
N3—C7—N4	121.0 (3)	N9—C25—N10	116.9 (3)
N4—C12—H12A	109.5	N9—C29—H29A	109.5
N4—C12—H12B	109.5	N9—C29—H29B	109.5
H12A—C12—H12B	109.5	H29A—C29—H29B	109.5
N4—C12—H12C	109.5	N9—C29—H29C	109.5
H12A—C12—H12C	109.5	H29A—C29—H29C	109.5
H12B—C12—H12C	109.5	H29B—C29—H29C	109.5
C13—N5—C17	120.3 (3)	N2—C4—C3	110.1 (3)
C13—N5—C14	122.9 (3)	N2—C4—H4A	109.6
C17—N5—C14	115.2 (3)	C3—C4—H4A	109.6
C13—N6—C18	120.9 (3)	N2—C4—H4B	109.6
C13—N6—C16	121.1 (3)	C3—C4—H4B	109.6
C18—N6—C16	117.6 (3)	H4A—C4—H4B	108.1
C7—N3—C11	120.4 (3)	N1—C2—C3	110.1 (3)
C7—N3—C8	122.3 (3)	N1—C2—H2A	109.6
C11—N3—C8	116.2 (3)	C3—C2—H2A	109.6
N5—C17—H17A	109.5	N1—C2—H2B	109.6
N5—C17—H17B	109.5	C3—C2—H2B	109.6
H17A—C17—H17B	109.5	H2A—C2—H2B	108.2
N5—C17—H17C	109.5	C22—C21—C20	109.9 (3)
H17A—C17—H17C	109.5	C22—C21—H21A	109.7
H17B—C17—H17C	109.5	C20—C21—H21A	109.7
O3—C13—N6	119.3 (3)	C22—C21—H21B	109.7
O3—C13—N5	120.2 (3)	C20—C21—H21B	109.7
N6—C13—N5	120.5 (3)	H21A—C21—H21B	108.2
N1—C5—H5A	109.5	N8—C24—H24A	109.5
N1—C5—H5B	109.5	N8—C24—H24B	109.5
H5A—C5—H5B	109.5	H24A—C24—H24B	109.5
N1—C5—H5C	109.5	N8—C24—H24C	109.5
H5A—C5—H5C	109.5	H24A—C24—H24C	109.5
H5B—C5—H5C	109.5	H24B—C24—H24C	109.5
N2—C6—H6A	109.5	N3—C11—H11A	109.5
N2—C6—H6B	109.5	N3—C11—H11B	109.5
H6A—C6—H6B	109.5	H11A—C11—H11B	109.5
N2—C6—H6C	109.5	N3—C11—H11C	109.5
H6A—C6—H6C	109.5	H11A—C11—H11C	109.5
H6B—C6—H6C	109.5	H11B—C11—H11C	109.5
C19—N7—C23	119.9 (3)	N10—C30—H30A	109.5
C19—N7—C20	124.1 (3)	N10—C30—H30B	109.5
C23—N7—C20	115.4 (3)	H30A—C30—H30B	109.5
C25—N10—C28	124.9 (3)	N10—C30—H30C	109.5
C25—N10—C30	119.2 (3)	H30A—C30—H30C	109.5
C28—N10—C30	115.9 (3)	H30B—C30—H30C	109.5
N7—C20—C21	110.6 (3)	C10—C9—C8	109.4 (3)
N7—C20—H20A	109.5	C10—C9—H9A	109.8
C21—C20—H20A	109.5	C8—C9—H9A	109.8
N7—C20—H20B	109.5	C10—C9—H9B	109.8
C21—C20—H20B	109.5	C8—C9—H9B	109.8
H20A—C20—H20B	108.1	H9A—C9—H9B	108.2
N6—C18—H18A	109.5	N4—C10—C9	110.6 (3)
N6—C18—H18B	109.5	N4—C10—H10A	109.5
H18A—C18—H18B	109.5	C9—C10—H10A	109.5
N6—C18—H18C	109.5	N4—C10—H10B	109.5
H18A—C18—H18C	109.5	C9—C10—H10B	109.5
H18B—C18—H18C	109.5	H10A—C10—H10B	108.1
N5—C14—C15	110.9 (3)	N8—C22—C21	109.6 (3)
N5—C14—H14A	109.5	N8—C22—H22A	109.7
C15—C14—H14A	109.5	C21—C22—H22A	109.7
N5—C14—H14B	109.5	N8—C22—H22B	109.7
C15—C14—H14B	109.5	C21—C22—H22B	109.7
H14A—C14—H14B	108.0	H22A—C22—H22B	108.2
N3—C8—C9	109.7 (3)	N7—C23—H23A	109.5
N3—C8—H8A	109.7	N7—C23—H23B	109.5
C9—C8—H8A	109.7	H23A—C23—H23B	109.5
N3—C8—H8B	109.7	N7—C23—H23C	109.5
C9—C8—H8B	109.7	H23A—C23—H23C	109.5
H8A—C8—H8B	108.2	H23B—C23—H23C	109.5
C16—C15—C14	109.0 (3)	Al1—O6—Al1^i^	103.84 (12)
C16—C15—H15A	109.9	Al1—O6—H6	128 (4)
C14—C15—H15A	109.9	Al1^i^—O6—H6	127 (4)
			
O2—Al1—O1—C1	−133.5 (4)	C17—N5—C14—C15	−174.3 (3)
O6—Al1—O1—C1	39.4 (4)	C7—N3—C8—C9	−32.0 (4)
O3—Al1—O1—C1	132.8 (4)	C11—N3—C8—C9	160.2 (3)
O6^i^—Al1—O1—C1	−40.5 (4)	N5—C14—C15—C16	−49.1 (4)
Al1^i^—Al1—O1—C1	−3.2 (4)	C25—N9—C26—C27	36.6 (4)
O1—Al1—O3—C13	117.8 (3)	C29—N9—C26—C27	−151.0 (3)
O2—Al1—O3—C13	13.4 (4)	C28—C27—C26—N9	−54.5 (4)
O6—Al1—O3—C13	−126.8 (3)	C24—N8—C19—O4	12.2 (5)
O6^i^—Al1—O3—C13	−87.8 (5)	C22—N8—C19—O4	170.1 (3)
Al1^i^—Al1—O3—C13	−118.8 (3)	C24—N8—C19—N7	−169.3 (3)
Al1—O1—C1—N2	−94.1 (4)	C22—N8—C19—N7	−11.4 (5)
Al1—O1—C1—N1	88.0 (4)	C23—N7—C19—O4	−6.2 (5)
C6—N2—C1—O1	−0.1 (4)	C20—N7—C19—O4	−176.9 (3)
C4—N2—C1—O1	−176.1 (3)	C23—N7—C19—N8	175.3 (3)
C6—N2—C1—N1	177.7 (3)	C20—N7—C19—N8	4.6 (5)
C4—N2—C1—N1	1.7 (4)	C13—N6—C16—C15	−39.2 (4)
C5—N1—C1—O1	−2.8 (4)	C18—N6—C16—C15	147.6 (3)
C2—N1—C1—O1	178.6 (3)	C14—C15—C16—N6	57.8 (4)
C5—N1—C1—N2	179.4 (3)	C25—N10—C28—C27	−15.1 (5)
C2—N1—C1—N2	0.8 (4)	C30—N10—C28—C27	165.3 (3)
O1—Al1—O2—C7	−104.2 (5)	C26—C27—C28—N10	44.8 (4)
O6—Al1—O2—C7	86.0 (5)	C26—N9—C25—O5	174.5 (3)
O3—Al1—O2—C7	−10.9 (5)	C29—N9—C25—O5	2.3 (5)
O6^i^—Al1—O2—C7	154.2 (5)	C26—N9—C25—N10	−5.6 (5)
Al1^i^—Al1—O2—C7	129.0 (5)	C29—N9—C25—N10	−177.9 (3)
Al1—O2—C7—N3	−101.8 (5)	C28—N10—C25—O5	173.8 (4)
Al1—O2—C7—N4	79.3 (6)	C30—N10—C25—O5	−6.6 (6)
C12—N4—C7—O2	11.5 (4)	C28—N10—C25—N9	−6.1 (5)
C10—N4—C7—O2	176.0 (3)	C30—N10—C25—N9	173.6 (3)
C12—N4—C7—N3	−167.3 (3)	C1—N2—C4—C3	23.8 (4)
C10—N4—C7—N3	−2.8 (4)	C6—N2—C4—C3	−152.4 (3)
O2—C7—N3—C11	−6.0 (4)	C2—C3—C4—N2	−50.2 (4)
N4—C7—N3—C11	172.9 (3)	C1—N1—C2—C3	−28.5 (4)
O2—C7—N3—C8	−173.3 (3)	C5—N1—C2—C3	152.8 (3)
N4—C7—N3—C8	5.6 (4)	C4—C3—C2—N1	52.6 (4)
Al1—O3—C13—N6	−117.6 (3)	N7—C20—C21—C22	49.4 (5)
Al1—O3—C13—N5	63.2 (4)	N3—C8—C9—C10	53.8 (4)
C18—N6—C13—O3	2.6 (4)	C7—N4—C10—C9	27.0 (4)
C16—N6—C13—O3	−170.4 (3)	C12—N4—C10—C9	−167.7 (3)
C18—N6—C13—N5	−178.1 (3)	C8—C9—C10—N4	−51.7 (4)
C16—N6—C13—N5	8.9 (4)	C19—N8—C22—C21	37.6 (5)
C17—N5—C13—O3	15.7 (4)	C24—N8—C22—C21	−163.8 (3)
C14—N5—C13—O3	−179.4 (3)	C20—C21—C22—N8	−55.5 (5)
C17—N5—C13—N6	−163.6 (3)	O1—Al1—O6—Al1^i^	−96.12 (13)
C14—N5—C13—N6	1.3 (4)	O2—Al1—O6—Al1^i^	73.02 (19)
C19—N7—C20—C21	−24.8 (5)	O3—Al1—O6—Al1^i^	170.23 (12)
C23—N7—C20—C21	164.2 (4)	O6^i^—Al1—O6—Al1^i^	0.000 (1)
C13—N5—C14—C15	20.1 (4)		

Symmetry code: (i) −*x*, −*y*+1, −*z*+1.

### Hydrogen-bond geometry (Å, º)

**Table:**

*D*—H···*A*	*D*—H	H···*A*	*D*···*A*	*D*—H···*A*
O6—H6···O4^i^	0.73 (5)	1.91 (5)	2.625 (3)	167 (5)
C5—H5*B*···I2	0.98	3.01	3.987 (3)	172
C6—H6*B*···O5^ii^	0.98	2.21	3.190 (4)	174
C12—H12*A*···O1	0.98	2.59	3.561 (4)	173
C12—H12*B*···I1^iii^	0.98	3.09	4.051 (3)	167
C14—H14*A*···I2^iv^	0.99	3.15	4.070 (4)	156
C17—H17*B*···I1^iv^	0.98	3.05	4.015 (4)	169
C16—H16*A*···I1^iii^	0.99	3.11	3.932 (4)	141
C24—H24*A*···O3^i^	0.98	2.57	3.482 (5)	154
C28—H28*B*···I2^v^	0.99	3.09	3.981 (4)	150
C30—H30*A*···O5^vi^	0.98	2.57	3.404 (5)	143

Symmetry codes: (i) −*x*, −*y*+1, −*z*+1; (ii) −*x*+1, −*y*+1, −*z*+2; (iii) *x*−1/2, −*y*+3/2, *z*+1/2; (iv) *x*−1, *y*, *z*; (v) *x*+1/2, −*y*+3/2, *z*+1/2; (vi) −*x*+2, −*y*+1, −*z*+2.
